# Transrectal versus transperineal prostate fusion biopsy: a pair-matched analysis to evaluate accuracy and complications

**DOI:** 10.1007/s00345-024-05245-1

**Published:** 2024-09-25

**Authors:** Marco Oderda, Romain Diamand, Rawad Abou Zahr, Julien Anract, Gregoire Assenmacher, Nicolas Barry Delongchamps, Alexandre Patrick Bui, Daniel Benamran, Giorgio Calleris, Charles Dariane, Mariaconsiglia Ferriero, Gaelle Fiard, Fayek Taha, Alexandre Fourcade, Georges Fournier, Karsten Guenzel, Adam Halinski, Giancarlo Marra, Guillaume Ploussard, Katerina Rysankova, Jean-Baptiste Roche, Giuseppe Simone, Olivier Windisch, Paolo Gontero

**Affiliations:** 1 Division of Urology, Department of Surgical Sciences, Molinette Hospital, University of Turin, Turin, Italy; 2 Department of Urology, Jules Bordet Institute-Erasme Hospital, Hôpital Universitaire de Bruxelles, Université Libre de Bruxelles, Brussels, Belgium; 3 Department of Urology, Cliniques Universitaires Saint-Luc, Brussels, Belgium; 4 Division of Urology, Cochin Hospital, APHP, Paris Cité University, Paris, France; 5 Department of Urology, Cliniques de L’Europe-Saint Elisabeth, Brussels, Belgium; 6 Department of Urology, Centre Hospitalier Universitaire de Reims, Reims, France; 7 Division of Urology, Geneva University Hospitals, Geneva, Switzerland; 8 Department of Urology, Hôpital Européen Georges-Pompidou, Université de Paris, Paris, France; 9 Department of Urology, IRCCS Regina Elena National Cancer Institute, Rome, Italy; 10 Department of Urology, Grenoble Alpes University Hospital, Université Grenoble Alpes, CNRS, Grenoble INP, TIMC, Grenoble, France; 11 Department of Urology, Hôpital Cavale Blanche, CHRU Brest, Brest, France; 12 Department of Urology, Vivantes Klinikum Am Urban, Berlin, Germany; 13 Department of Urology, Private Medical Center, Klinika Wisniowa”, Zielona Góra, Poland; 14 Department of Urology, La Croix du Sud Hospital, Quint Fonsegrives, France; 15 Department of Urology and Surgical Studies, Faculty of Medicine, University Hospital Ostrava, Ostrava University, Ostrava, Czech Republic; 16 Department of Urology, Clinique Saint-Augustin, Bordeaux, France

**Keywords:** Prostate biopsy, Fusion, Transrectal, Transperineal, Comparison, Complications, Accuracy

## Abstract

**Purpose:**

To evaluate biopsy-related complications and detection rates of any PCa and clinically significant PCa (csPCa, intended as grade group ≥ 2) between MRI-targeted TP fusion biopsies (TPBx) and TR ones (TRBx).

**Methods:**

We performed a multicentric study on 4841 patients who underwent fusion biopsy between 2016 and 2023. A case–control matching was performed to find comparable cohorts of 646 TPBx and 646 TRBx. Mean T test and Pearson chi-square tests were used to compare continuous and categorical variables.

**Results:**

Baseline characteristics were comparable between the cohorts, except for target location with a higher rate of anterior lesions in TPBx group. Complications were rare and no difference was found between the groups, with similar rates of infections after TRBx and TPBx (N = 5 (0.8%) vs N = 2 (0.3%), p 0.45). All patients in TRBx and 90.1% in TPBx group received antibiotic prophylaxis. A higher csPCa detection rate was found in TPBx over the group (50.5% vs 36.2%, p < 0.001). On average, positive targeted cores were increased in TPBx group, for any PCa (1.6 vs 1.4, p 0.04) and csPCa (1.0 vs 0.8, p 0.02). Among the limitations of study, we acknowledge the retrospective design and the possible under-reporting of complications.

**Conclusions:**

MRI-targeted fusion TPBx achieves a significantly higher csPCa detection than TRBx, with a diagnostic advantage for apical and anterior lesions. No significant differences were found in terms of complications that were rare in both groups, considering a widespread adoption of antibiotic prophylaxis.

## Introduction

In the last years there has been a shift from the transrectal (TR) to the transperineal (TP) approach for prostate biopsies. Current guidelines of the European Association of Urology recommend TP systematic biopsies over TR ones due to the lower risk of infectious complications [1, 2]. The TP route is also preferred for the detection of clinically significant prostate cancer (csPCa), especially for locations at the apex and in the anterior region [1, 3, 4]. Furthermore, the TP approach can be better in case of a subsequent focal therapy such as cryotherapy, irreversible electroporation, or targeted microwaves ablation, which can be performed only transperineally [5].

Nevertheless, the debate is still open: several observational and retrospective studies showed similar detection rates and complications between the two approaches [6–8], and a pair-matched comparison found comparable rates of csPCa, despite cancer length and cancer core involvement were in favour of TP biopsies (TPBx) [9]. The American Association of Urology (AUA) guidelines state that clinicians may use either a transrectal or transperineal biopsy route when performing a biopsy [10]. Recently, the ProBE-PC randomized trial failed to demonstrate any difference in the infectious or non-infectious complications among men undergoing TP or TR biopsies (TRBx) [11]. The PERFECT trial did not demonstrate noninferiority of TP over TR for MRI-targeted biopsy for significant PCa detection, despite a comparable overall detection rate for any-grade PCa [12]. Finally, the TRANSLATE trial is still underway to analyse the diagnostic ability of these two techniques [13].

The question is, what happens in the everyday clinical practice? Both TPBx and TRBx continue to be performed. We gathered data from a large, contemporary, multicentric series of patients who underwent TP or TR MRI-targeted and systematic prostate biopsy and performed a pair-matched analysis to compare the two approaches in terms of complications and csPCa detection.

## Patients and methods

### Study population

**Fig. 1:**
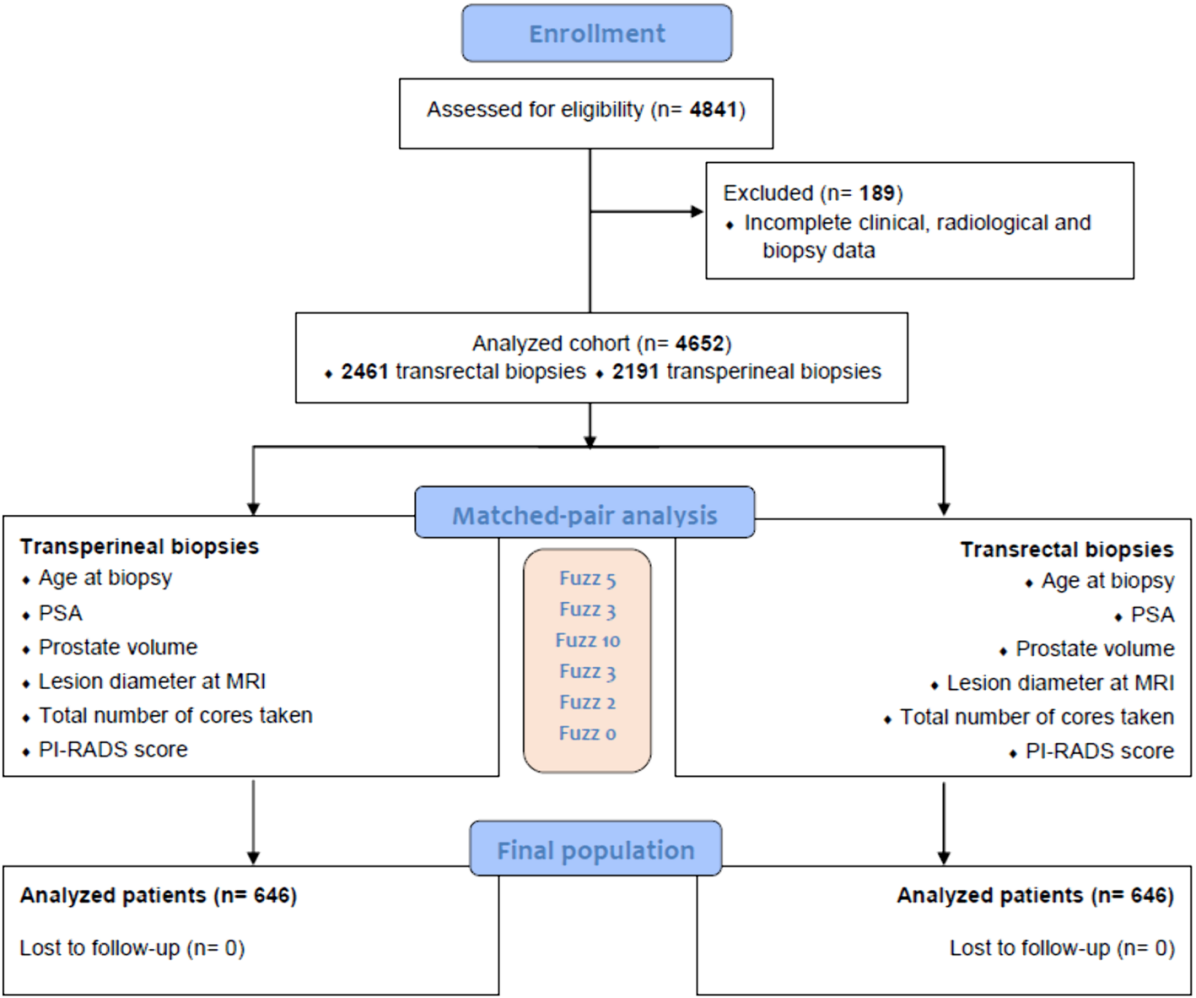
Study flowchart

Data from 4841 patients who underwent MRI-targeted and systematic prostate biopsy between January 2016 and April 2023 were retrospectively identified from an independent prospectively maintained databases at fifteen European tertiary referral-centers. Among these, we excluded patients with incomplete clinical, radiological and biopsy data, resulting in a final population of 4652 patients (Fig. 1). The study was conducted according to Helsinki declaration and all patients signed an informed consent for data collection. No formal ethical committee approval was needed according to the Agenzia Italiana del Farmaco—AIFA guidelines for observational studies.

### MRI and biopsy technique

All prebiopsy MRIs were performed using a 1.5-T or 3-T scanner, with or without an endorectal coil, and consisted of multiplane T1- and T2-weighted imaging, diffusion-weighted imaging (DWI), and dynamic contrast enhancement according to the European Society of Urogenital Radiology guidelines. Scans were reviewed and scored by experienced radiologists using the PI-RADS v.2 or 2.1 protocols. All MRIs had at least one lesion suspicious for PCa, defined as PI-RADS score ≥ 3.

All MRI-targeted prostate biopsies were performed under local anesthesia with the Koelis fusion imaging system (Koelis®, La Tronche, France) using the Urostation® (n = 90) or Trinity® (n = 4563) software platform. A minimum of 2 targeted cores per target were taken, followed by systematic biopsies. The number of systematic cores was chosen according to the urologist’s preference and prostate volume. Koelis system retraces the trajectory of every biopsy, allowing to check the position of the systematic mapping.

### Endpoints and statistical analyses

The endpoints of study were the evaluation of the biopsy-related complications and the detection of of any PCa and csPCa on targeted, systematic, and targeted + systematic sampling, comparing TR versus TP approach. csPCa was defined as ISUP grade group 2 or greater. Case–control matching was performed to find comparable cohorts of transrectal and transperineal biopsies from the original series of 4652 patients. The matching was performed using the following variables: age at biopsy (fuzz 5), PSA (fuzz 3), prostate volume (fuzz 10), lesion diameter at MRI (fuzz 3), total number of cores taken (fuzz 2), and PI-RADS score (fuzz 0). Continuous data were reported as means and standard deviation and categorical parameters were shown as counts/percentages. Mean T test and Pearson chi-square tests were used when appropriate to compare continuous and categorical variables. Statistical significance was set at two-sided p < 0.05. Statistical analyses were performed with SPSS version 28.0 (IBM Corp, Armonk, NY, USA).

## Results

From the original cohort of 4652 patients, 646 TP biopsies were pair-matched 1:1 with 646 TR biopsies. Baseline clinical and MRI characteristics, shown in Table 1, were comparable between the cohorts, except for target side and target location, which was more frequently anterior in the TPBx group (37.1% vs 25.8%) and posterior in the TRBx group (72.7% vs 58.0%). Also, there were significantly more patients with a prior negative biopsy in the TPBx group (18.9% vs 2.2%). No significant differences were reported between the two groups in terms of age, PSA, PSA-density, positive digital rectal examination (DRE), prostate volume at MRI, number or size of suspicious lesions at MRI, and PI-RADS score.

**Table 1:** Baseline data

	Overall	Transrectal	Transperineal	p
Number of patients	1292	646	646	-
Age, yrs, mean (SD)	66.9 (7.1)	66.8 (7.0)	67.1 (7.2)	0.46
PSA, ng/ml, mean (SD)	7.0 (3.4)	7.1 (3.3)	6.9 (3.4)	0.46
PSA density at RP, mean (SD)	0.16 (0.1)	0.16 (0.1)	0.15 (0.1)	0.43
Positive DRE, n (%)	269 (20.9%)	150 (23.3%)	119 (18.5%)	0.04
Previous negative biopsy, n (%)	136 (10.5%)	14 (2.2%)	122 (18.9%)	< 0.001
Multiparametric MRI				
Prostate volume at mpMRI, cc, mean (SD)	49.6 (21.9)	49.6 (22.0)	49.5 (21.8)	0.95
Number of suspicious lesions, n (%)				0.79
- single	987 (76.4%)	491 (76.0%)	496 (76.8%)	
- multiple	305 (23.6%)	155 (24.0%)	150 (23.2%)	
PI-RADS score, n (%)				0.08
- 3	226 (17.5%)	98 (15.2%)	128 (19.8%)	
- 4	797 (61.7%)	410 (63.5%)	387 (59.9%)	
- 5	269 (20.8%)	138 (21.4%)	131 (20.3%)	
Target diameter, mm, mean (SD)	11.7 (4.7)	11.8 (4.6)	11.5 (4.8)	0.23
Target side, n (%)*				< 0.00
- left	582 (45.6%)	281 (43.8%)	301 (47.3%)	1
- right	496 (38.8%)	277 (43.2%)	219 (34.4%)	
- middle	32 (2.5%)	24 (3.7%)	8 (1.3%)	
- bilateral	167 (13.1%)	59 (9.2%)	108 (17.0%)	
Target location, n (%)**				< 0.001
- anterior	391 (31.4%)	163 (25.8%)	228 (37.1%)	
- posterior	816 (65.5%)	459 (72.7%)	357 (58.0%)	
- transitional	39 (3.1%)	9 (1.4%)	30 (4.9%)	
Target location, n (%) #				0.18
- apex	390 (31.1%)	189 (30.2%)	201 (31.9%)	
- equator	485 (38.6%)	252 (40.3%	233 (37.0%)	
- base	268 (21.4%)	139 (22.2%	129 (20.5%)	
- apex/equator	44 (3.5%)	16 (2.6%)	28 (4.4%)	
- base/equator	68 (5.4%)	29 (4.6%)	39 (6.2%)	

Legend: DRE = digital rectal examination; ISUP GG = International Society of Urological Pathology; mpMRI = multiparametric Magnetic Resonance Imaging; PI-RADS = Prostate Imaging Reporting & Data System; HGPIN = High-Grade Prostatic Intraepithelial Neoplasia. *missing location in 15 cases; **missing location in 46 cases; #missing location in 37 cases

### Complications

Complications were rare and no significant differences were found between the groups, as shown in Table 2. All patients in the TRBx and 90.1% in the TPBx group received antibiotic prophylaxis. Post-biopsy infections were recorded in only 7 patients, with similar rates after TRBx and TPBx (N = 5 (0.8%) vs N = 2 (0.3%), p 0.45). Acute urinary retention was comparable between groups, with 3 cases per group. Mild bleeding complications, including persistent macrohematuria, hemospermia or rectorrhagia (the latter for TRBX) were similar between groups and resolved spontaneously.

**Table 2:** Biopsy complications

	Overall	Transrectal	Transperineal	p
Antibiotic prophylaxis, n (%)	1228 (95.0%)	646 (100.0%)	582 (90.1%)	< 0.001
Urinary tract infection needing medical treatment and/or hospitalization	7 (0.5%)	5 (0.8%)	2 (0.3%)	0.45
Persistent dysuria without signs of UTI	4 (0.3%)	2 (0.3%)	2 (0.3%)	1.00
Bleeding, including persistent macrohematuria, hemospermia or rectorrhagia	17 (1.3%)	8 (1.2%)	9 (1.4%)	1.00
Acute urinary retention	6 (0.5%)	3 (0.5%)	3 (0.5%)	1.00
Vagal crisis during biopsy	1 (0.1%)	1 (0.2%)	0 (0.0%)	0.12

*Reported percentages consider missing values in each category

### PCa detection rate

All biopsy results are shown in Table 3. A higher csPCa detection rate was found in the TPBx over the TRBx group (50.5% vs 36.2%, p < 0.001), whereas no statistical difference was found for the overall PCa detection rate (68.0% vs 70.3%, p 0.39. When considering only targeted cores, TPBx achieved higher detection of both csPCa (44.0% vs 30.5%, p < 0.001) and any PCa (60.4% vs 54.6%, p 0.04). On average, positive targeted cores were increased in the TPBx group, for any PCa (1.6 vs 1.4, p 0.04) and csPCa (1.0 vs 0.8, p 0.02). When considering systematic biopsies only, PCa detection rate was higher for TRBx (59.4% vs 43.5%, p < 0.001) but there was no difference in csPCa detection.

**Table 3:** Cancer detection rates of targeted and systematic biopsies

	Overall	Transrectal	Transperineal	P
Number of patients	1292	646	646	-
Prostate biopsy (targeted + systematic)				
Total number of cores taken, mean (SD)	13.8 (3.1)	13.8 (2.9)	13.7 (3.3)	0.71
Cancer detection rate, n (%)	893 (69.1%)	454 (70.3%)	439 (68.0%)	0.39
csPCa detection rate, n (%)	560 (43.3%)	234 (36.2%)	326 (50.5%)	< 0.001
ISUP grade, n (%)				< 0.001
- 1	333 (37.3%)	220 (48.5%)	113 (25.7%)	
- 2	309 (34.6%)	129 (28.4%)	180 (41.0%)	
- 3	123 (13.8%)	42 (9.3%)	81 (18.5%)	
- 4	106 (11.9%	54 (11.9%)	52 (11.8%)	
- 5	22 (2.5%)	9 (2.0%)	13 (3.0%)	
Targeted only				
Targeted cores taken, mean (SD)	3.8 (1.4)	3.7 (1.4)	3.9 (1.4)	0.009
Positive targeted cores, mean (SD)	1.5 (1.6)	1.4 (1.6)	1.6 (1.7)	0.04
Positive targeted cores ISUP ≥ 2, mean (SD)	0.9 (1.5)	0.8 (1.4)	1.0 (1.5)	0.02
Cancer detection rate, n (%)	743 (57.5%)	353 (54.6%)	390 (60.4%)	0.04
csPCa detection rate, n (%)	481 (37.2%)	197 (30.5%)	284 (44.0%)	< 0.001
csPCa location on target, n (%)*				< 0.001
- anterior	130 (33.2%)	40 (24.5%)	90 (39.5%)	0.002
- posterior	308 (33.7%)	152 (33.1%)	156 (43.1%)	0.002
- transitional	18 (46.2%)	2 (22.2%)	16 (53.3%)	0.13
csPCa location on target, n (%)**				< 0.001
- apex	156 (40.0%)	66 (34.9%)	90 (44.8%)	0.05
- equator	187 (38.6%)	78 (31.0%)	109 (46.8%)	< 0.001
- base	79 (29.5%)	27 (19.4%)	52 (40.3%)	< 0.001
- apex/equator	15 (34.1%)	8 (50.0%)	7 (25.0%)	0.11
- base/equator	9 (42.6%)	11 (37.9%)	18 (46.2%)	0.62
ISUP grade, n (%)				< 0.001
- 1	262 (35.3%)	156 (44.2%)	106 (27.2%)	
- 2	270 (36.3%	118 (33.4%	152 (39.0%)	
- 3	110 (14.8%)	) 36 (10.2%)	74 (19.0%)	
- 4	80 (10.8%)	34 (9.6%)	46 (11.8%)	
- 5	21 (2.8%)	9 (2.5%)	12 (3.1%)	
Systematic only				
Systematic cores taken, mean (SD)	10.0 (2.8)	10.1 (2.6)	9.8 (3.0)	0.08
Positive systematic cores, mean (SD)	1.5 (2.1)	1.8 (2.3)	1.2 (1.9)	< 0.001
Positive systematic cores ISUP ≥ 2, mean (SD)	0.7 (1.5)	0.7 (1.5)	0.7 (1.5)	0.39
Cancer detection rate, n (%)	665 (51.5%)	384 (59.4%)	281 (43.5%)	< 0.001
csPCa detection rate, n (%)	361 (27.9%)	173 (26.8%)	188 (29.1%)	0.38
ISUP grade, n (%)				< 0.001
- 1	304 (45.7%)	211 (54.9%)	93 (33.1%)	
- 2	223 (33.5%)	104 (27.1%)	119 (42.3%)	
- 3	66 (9.9%)	25 (6.5%)	41 (14.6%)	
- 4	61 (9.2%)	37 (9.6%)	24 (8.5%)	
- 5	11 (1.7%)	7 (1.8%)	4 (1.4%)	

*Missing location in 25 cases; **missing location in 15 cases

TPBx achieved higher csPCa detection rate over TRBx in both anterior (39.5% vs 24.5%, p 0.002) and posterior lesions (43.1% vs 33.1%, p 0.002), and in targets located at the apex (44.8% vs 34.9%, p 0.05), the equatorial region (46.8% vs 31.0%, p < 0.001), and the base (40.3% vs 19.4%, p < 0.001).

### Subgroup analysis

TPBx achieved higher csPCa detection rate over TRBx in both biopsy-naïve patients (51.1% vs 36.7%, p < 0.001) and in the 136 cases with previous negative prostate biopsies (47.5% vs 14.2%, p 0.02). When focusing on the number of targeted lesions, TPBx achieved higher csPCa detection rate over TRBx in unilateral lesions (51.5% vs 35.0%, p < 0.001); csPCa detection was similar between TPBx and TRBx in the 167 cases where lesions were bilateral (44.4% vs 49.1%, p 0.62).

## Discussion

In the last years, the TP approach for prostate biopsy has become recommended by the European Association of Urology [1] due to several reasons: it can be performed under local anaesthesia with excellent tolerability [14]; it is a quick procedure with no impact on erectile or urinary function [15]; acute urinary retentions remain a rare event, more frequent in huge glands [15, 16]; a better csPCa detection has been suggested as compared to TRBx, especially in case of apical or anterior lesions [3, 4, 17]; last but not least, the lower infection rates, with a meta-analysis of 7 studies (1.330 participants) showing a relative risk of 0.55 (95% CI 0.33–0.92, p 0.02) as compared to TRBx [2]. On the other hand, the American Association of Urology guidelines do not express a preference between the TR and the TP approach [10], in line with the results of several randomized trials that did not show significant differences between these two techniques [11, 12, 18]. The PREVENT trial randomized 658 participants, with zero TP versus four TR biopsy infections and similar detection of csPCa (53% TP versus 50% TR) [18]. The ProBE-PC study also randomized 840 patients, with csPCa detection rates of 47% and 43% for TR and TP biopsy, respectively [19]. Also, different retrospective studies reported similar complication rates, including infections, and no significant differences in cancer detection rate between the two approaches [6–8]. A pair-matched analysis by Kaneko (168 fusion TPBx versus 336 fusion TRBx) showed similar csPCa detection (per patient and per lesion), despite larger cancer core length and percent of core involvement were in favour of TPBx [9].

In the present study, we analysed a large, multicentric series of patients undergoing TR or TP fusion biopsies with the same device and the same protocol, including targeted and systematic cores. Despite its retrospective nature, the comparison was controlled by a case–control matching for the main variables of interest that might impact on complications (age, prostate volume, number of cores) or detection rates (PSA, lesion diameter, PI-RADS score).

Concerning the biopsy-related complications, we did not find any significant difference between the two groups, in line with the results of the ProBE-PC randomized trial that failed to demonstrate any difference in the infectious or non-infectious complications [11]. Also, the recent PREVENT trial, which randomized 658 patients to TPBx without antibiotic prophylaxis versus TRBx with targeted prophylaxis based on rectal cultures, found very low and similar rates of infections (0 in TPBx versus 4 in TRBx) and other complications [18]. We acknowledge the difficulty to routinely use rectal cultures in the everyday clinical practice, and to completely abandon the antibiotic prophylaxis in TPBx. The NORAPP randomized trial had shown that the rates of infections are not higher in patients not receiving antibiotic prophylaxis before TPBx than in those receiving it, suggesting that antibiotic prophylaxis might be omitted in this population [20]. Nevertheless, antibiotics are still greatly prescribed to patients undergoing biopsy, as demonstrated in our study by the 100% and 90.1% in the TRBx and TPBx groups, respectively. The retrospective design of our study might be responsible for the under-reporting of complications, which were very low in both groups. Recently, the PERFECT randomized trial found no difference in adverse events of grade ≥ 2 between TP (35.7%) and TR (40.5%, p 0.4) biopsies, with only one TR patient experiencing grade 3 sepsis [12].

Concerning the csPCa detection rate, we found it significantly higher in the TP approach over the TR one, in line with the findings of a recent multicenter retrospective analysis that showed a higher detection of csPCa in TPBx (n = 3.305) as compared to TRBx (n = 1.936) [4]. The TPBx group achieved a higher csPCa detection despite having significantly more patients with a prior negative biopsy, which should reduce the rate of biopsy positivity according to a recent study [21]. In our study, the TP targeted cores achieved a higher detection of both csPCa and any PCa, probably due to the ease of sampling anterior and apical lesions. Zattoni et al. had already described that TPBx have a significantly higher likelihood than TRBx to detect csPCa in the apex (OR 4.81) and anterior zone (OR 5.62) [4]. It was suggested that the adoption of TPBx compared with TRBx may reduce the risk of upgrading and improve the concordance of biopsy grade with the final pathology [22]. The advantage in the detection of anterior (OR 2.17) and apical (OR 1.86) tumors was also confirmed in a recent meta-analysis gathering data from 3.522 and 5.140 patients who underwent, respectively, TR and TP MRI-targeted biopsies [17]. Our study found an advantage for TPBx also in the detection of posterior lesions, and those located at the equator or the base of the prostate. We can only hypothesize that TPBx could sample a higher amount of tissue in the posterior zone as compared to TRBx due to the angle of insertion of the biopsy needle, leading to an increased detection. However, when speaking of accuracy of prostate biopsies, we need to consider a certain number of false positives at MRI that could be differently allocated in the the TRBx and TPBx groups.

The recently published PERFECT trial did not demonstrate the noninferiority of TP over TR biopsies for significant PCa detection, with ISUP ≥ 2 detection of 47.2% in the TP group as compared with 54.2% in the TR group (p 0.6, 2% under the statistical margin for noninferiority) [12]. These results might be affected by the higher rate of PIRADS 5 lesions and secondary PIRADS 4–5 lesions in the TR group in spite of randomization. Also, the different experience of each center in TR or TP biopsies might have played a role. The TRANSLATE trial is currently underway, being 90% powered to detect a 10% difference between the two techniques, with TPBx hypothesized at 55% detection rate for csPCa versus 45% for TRBx [13]. Interestingly, our study showed that TR systematic mapping detected higher rates of PCa as compared to TP one, but mostly ISUP 1 (54.9% vs 33.1%). It is hard to find an explanation for such a difference that has little clinical impact, given that ISUP 1 PCa is not considered clinically significant.

As previously said, our study is not devoid of limitations, mainly due to its retrospective design that might have underestimated the number of biopsy-related complications and brought a selection bias with more anterior lesions in the TPBx group and, conversely, more posterior lesions in the TRBx group. Nevertheless, the case–control matching and the large sample size allowed for a proper comparison of the two cohorts.

## Conclusions

MRI-targeted fusion TPBx achieved a significantly higher csPCa detection than TRBx, and targeted cores diagnosed more PCa and csPCa with the TP approach over the TR one. No significant differences were found in terms of complications that were rare in both groups, considering a widespread adoption of antibiotic prophylaxis.

## Data Availability

Data available on reasonable request.
